# Tumor cell-derived exosomes home to their cells of origin and can be used as Trojan horses to deliver cancer drugs

**DOI:** 10.7150/thno.39434

**Published:** 2020-02-10

**Authors:** Li Qiao, Shiqi Hu, Ke Huang, Teng Su, Zhenhua Li, Adam Vandergriff, Jhon Cores, Phuong-Uyen Dinh, Tyler Allen, Deliang Shen, Hongxia Liang, Yongjun Li, Ke Cheng

**Affiliations:** 1 Department of Molecular Biomedical Science, North Carolina State University, Raleigh, NC, USA.; 2 Department of Biomedical Engineering, University of North Carolina, Chapel Hill, and North Carolina State University, Raleigh, NC, USA.; 3 Department of Cardiology, The Second Hospital of Hebei Medical University, Shijiazhuang, China.; 4 Department of Infectious Diseases and Hepatology, The First Affiliated Hospital of Zhengzhou University, Zhengzhou, China.; 5 Department of Cardiology, The First Affiliated Hospital of Zhengzhou University, Zhengzhou, China.

**Keywords:** exosome, homing, integrin, doxorubicin, cancer therapy

## Abstract

Cancer is the second leading cause of death worldwide and patients are in urgent need of therapies that can effectively target cancer with minimal off-target side effects. Exosomes are extracellular nano-shuttles that facilitate intercellular communication between cells and organs. It has been established that tumor-derived exosomes contain a similar protein and lipid composition to that of the cells that secrete them, indicating that exosomes might be uniquely employed as carriers for anti-cancer therapeutics.

**Methods**: We isolated exosomes from two cancer cell lines, then co-cultured each type of cancer cells with these two kinds of exosomes and quantified exosome. HT1080 or Hela exosomes were systemically injected to Nude mice bearing a subcutaneous HT1080 tumor to investigate their cancer-homing behavior. Moreover, cancer cell-derived exosomes were engineered to carry Doxil (a common chemotherapy drug), known as D-exo, were used to detect their target and therapeutic efficacy as anti-cancer drugs. Exosome proteome array analysis were used to reveal the mechanism underly this phenomenon.

**Results**: Exosomes derived from cancer cells fuse preferentially with their parent cancer cells, in vitro. Systemically injected tumor-derived exosomes home to their original tumor tissues. Moreover, compared to Doxil alone, the drug-loaded exosomes showed enhanced therapeutic retention in tumor tissues and eradicated them more effectively in nude mice. Exosome proteome array analysis revealed distinct integrin expression patterns, which might shed light on the underlying mechanisms that explain the exosomal cancer-homing behavior.

**Conclusion**: Here we demonstrate that the exosomes' ability to target the parent cancer is a phenomenon that opens up new ways to devise targeted therapies to deliver anti-tumor drugs.

## Introduction

Cancer is a life-threating public health problem responsible for an estimated 9.6 million deaths worldwide in 2018 1. The most common treatment option available for cancer patients is chemotherapy. While chemotherapeutic drugs can inhibit the progression of cancers and even suppress cancer to the point of remission, they come with many serious side-effects. The systemic infusion of chemotherapy drugs can cause cardiomyopathy, vomiting, asthenia, and alopecia due to their inability to target the cancerous tissue of the body exclusively. Therefore, effective new targeted cancer treatments are urgently needed.

Tumor cells produce and secrete more nucleic acids, proteins, and lipids than healthy cells. Some of these molecules are transported in the blood or encapsulated in extracellular microvesicles, such as exosomes, that are released into the extracellular environment. Once released, these exosomes become a part of a communication network used by other tumor cells and organs 2-4. Tumor-derived exosomes contain an abundant biological content that resembles that of their parent tumor cell. These include DNAs, RNAs, transmembrane receptors, growth factors, angiogenic factors, and extracellular matrix (ECM) molecules 2. In addition, they store intercellular signaling messengers involved in the pathogenesis, development, progression, and metastasis of cancer 3, 4. Previous studies have proven the concept of delivering exosome-encapsulated molecules, including chemotherapeutics, to tumor sites. Tian Y et. al., for example, encapsulated doxorubicin in immature dendritic cell-derived exosomes for targeting αv integrin-positive cancer cells 5. Herein, we utilize the abundance and ease of acquisition of cancer-derived exosomes to create an even more effective targeted delivery platform.

To date, the signaling mechanisms involved in exosome release, transmission, and uptake have not been clearly described. Previous studies, including those from our lab, suggest that exosome can be absorbed by the parent cell line, as well as other cells of various origins 6-9. However, they seem to most readily interact with cancer cells, compared to normal cells, and it appears that this interaction is driven, at least in part, by lipid mixing 10-12.

Since exosomes are extracellular nano-shuttles that facilitate intercellular communication between cells, the constitution of exosomes, as well as their biological activity, is largely dependent on the demands of their parent cells. Tumor-derived exosomes contain a similar protein and lipid composition to that of the cells that secrete them, indicating that exosomes might be uniquely designed to interact with their parent cell line in comparison to other cells. Thus, we hypothesized that tumor cell-derived exosomes might be especially effective at traveling back to the parent cell line that produced them. If so, their exosomes could be used as Trojan horses that deliver anticancer drugs to the cancer cells. To test this idea, we investigated the biodistribution of tumor-derived exosomes, and profiled the proteome of exosomes from mouse tumor models. We found that exosomal integrins might direct the tumor-specific colonization of cancer exosomes. Remarkably, we found that the tropism exhibited by tumor-secreted exosomes can be utilized to shuttle Doxil, a cancer therapeutic with no specific targeting capacity, to tumor sites.

## Results

### Tumor exosomes home to their parent cells in vitro and in vivo

**Figure 1:**
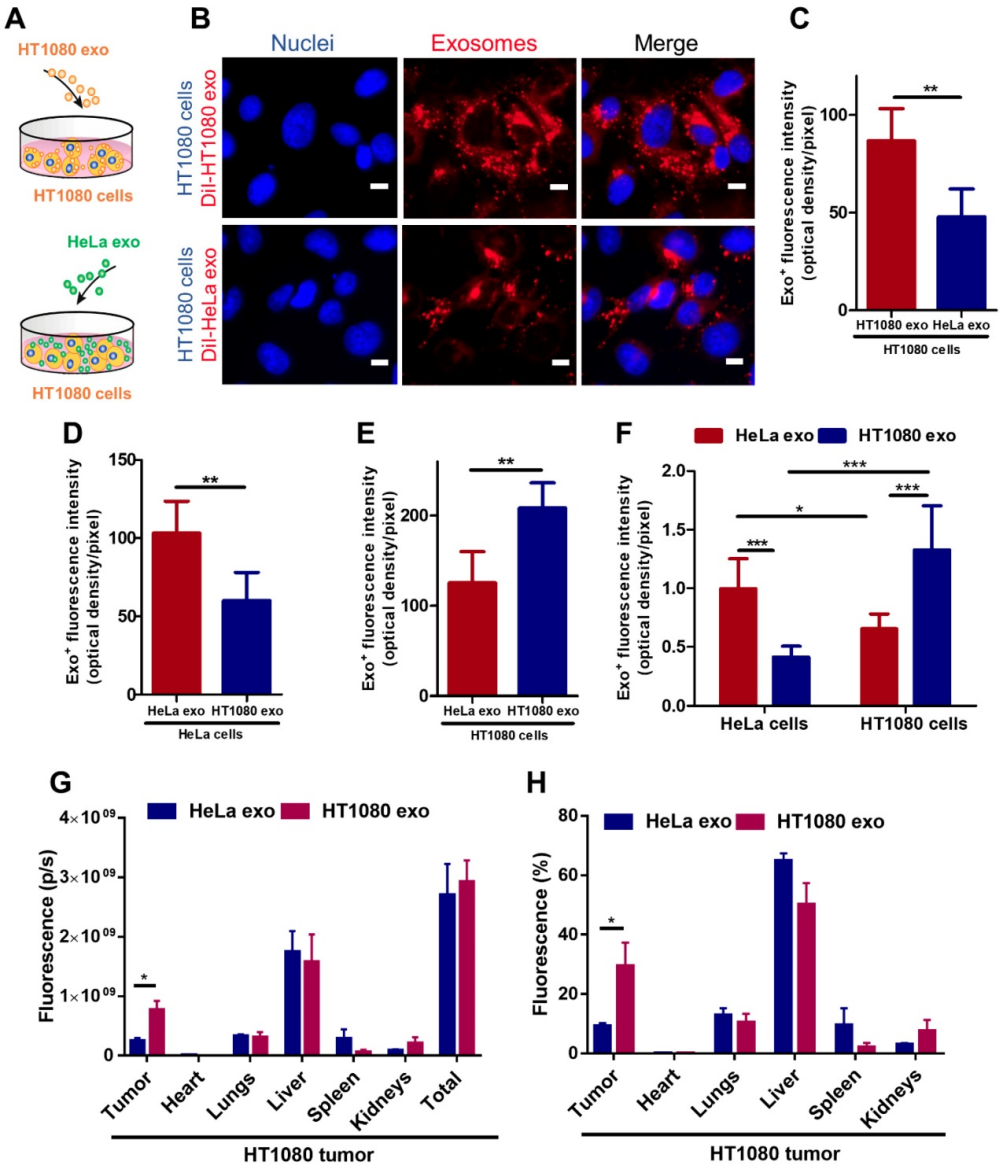
** Cancer cell derived exosomes preferably fuse with their parent cells.** (A) Schematic showing the in vitro study design to assess the homing ability of cancer exosomes. (B) Representative micrographs showing uptake of DiI-labeled HT1080 exosomes and HeLa exosomes by HT1080 cells. Endocytosed exosomes (red) can be seen around nuclei. Scale bar: 5µm. (C) Quantitation of exosomal uptake in (B) (n=6). (D-E) Quantitation of exosomal uptake in HeLa cells and HT1080 cells (n=6). (F) Quantitation of exosomal uptake by Microplate Fluorometer (n=10-12). (G-H) Exosomes biodistribution in vivo. p/s: photons/second. (G) Quantitation of fluorescence of DiR-labeled-exosomes in major organs and tumor tissues (n=3). (H) Quantitation of the percentage of exosomes in major organs and tumor tissue (n=3). (C-H) Two-tailed t-test. *, p<0.05. **, p<0.01. ***, p<0.001. All values are mean ± S.D.

To detect whether tumor exosomes home to their parent cells in vitro, we isolated exosomes from HT1080 (a fibrosarcoma cell line) and HeLa (a cervical cancer cell line) cell lines. Flow cytometry was used to detect common exosome markers: CD81, CD63 and CD9 (Figure S1). We then co-cultured HT1080 cells with DiI-labeled-HT1080 exosomes or Dil-labeled-HeLa exos for 12 hours and quantified exosome uptake in HT1080 cells using fluorescence microscopy, the labeling efficiency is higher than 90% (Figure S2). We observed twice as much uptake of HT1080 exosomes versus HeLa exosomes in HT1080 cells (Figures 1 A-C). To make sure this finding was not an HT1080-specific phenomenon, we tested whether exosomes from HeLa cells would also exhibit parental cell tropism. We co-cultured HeLa cells with red (DiI) fluorescently labeled HeLa exosomes and green (DiO) fluorescently labeled HT1080 exosomes. Although both types of exosomes were internalized in HeLa cells, there was significantly more HeLa exosome uptake than HT1080 exosome uptake. Interestingly, HT1080 cells absorbed HT1080 exosomes about twice as efficiently as HeLa cells absorbed HeLa exosomes (Figures 1 D-E; Figures S3A-B). Then, the dye labeling for the exosomes were reversed to ensure that the uptake differences are not due to differences in labeling (Figures S3C-D). Subsequently, we designed another in vitro test, using a quantitative fluorometer to validate these findings. HeLa and HT1080 cells were seeded in different wells and treated with exosomes derived from the same cell type or the other. HT1080 exosomes were three times more efficiently absorbed by HT1080 cells than HeLa cells, and HeLa exosomes were absorbed mainly by HeLa cells as opposed to HT1080 cells (Figure 1 F; Figure S3E). Therefore, the cell specificity of exosome localization matched the cell of origin of the exosome, suggesting a more efficient uptake of exosomes by parent cancer cells compared to cells of other origins.

To substantiate this finding in vivo, we created an HT1080 tumor model using immunodeficient nude mice, and intravenously injected them with DiR-labeled HT1080 or HeLa exosomes (Figures S4A-B). The exosomes' biodistribution was detected with an IVIS imaging system. HT1080 exosomes preferentially localized to tumor tissue. In fact, the expression of the fluorescent signal for HT1080 exosomes is more than three times that of the HeLa exosomes. By contrast, HeLa exosomes abundantly presented in major organs (64.86% in the liver) rather than colonizing the tumor site (Figures 1 G-H). Taken together, our data suggest that cancer exosomes preferentially home to their parent cancer cell of origin.

### D-exo fabrication and characterization

**Figure 2:**
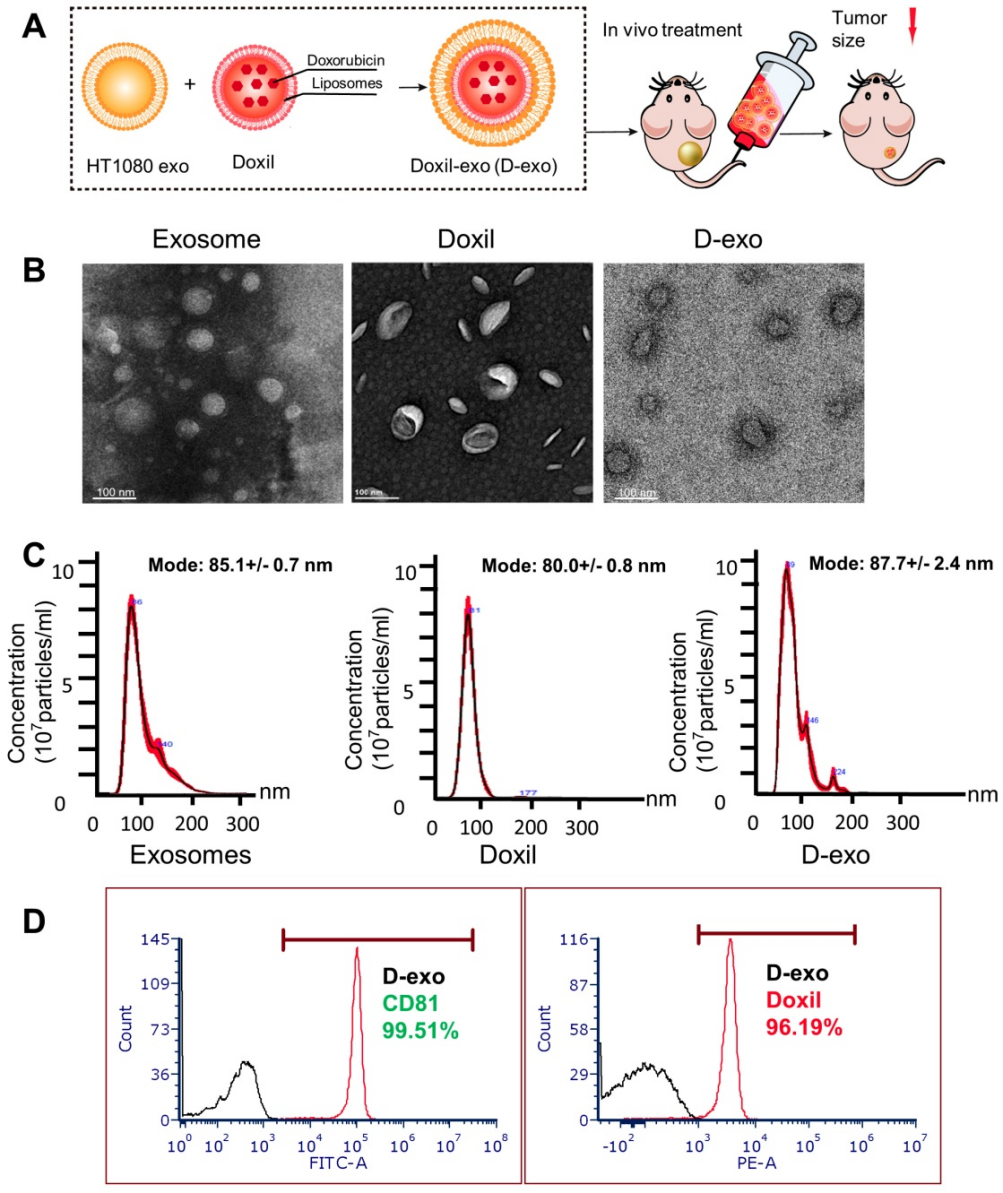
** Characterization of Doxil-exosomes (D-exo).** (A) Schematic illustration of the construction of drug-loaded D-exo and their effects in nude mice once intravenously injected. (B) Transmission electron microscopy (TEM) showing the morphology of exosomes, Doxil, and D-exo. (C) Size distribution of exosomes, Doxil, and D-exo attained from NanoSight particle tracking analysis (n=5). (D) Flow cytometry showing both exosomal marker, CD81, and Doxorubicin were positively expressed on D-exo, indicating the successful fabrication of D-exo.

Having established the homing ability of cancer exosomes, we hypothesized that anti-cancer drugs could be loaded into them and shuttled by them to tumor sites. We incorporated Doxil, an anti-cancer chemotherapy drug that encapsulates doxorubicin in a closed lipid sphere, into HT1080 exosomes via membrane extrusion (Figure 2A). Transmission electron microscopy (TEM) showed the morphology of exosomes, Doxil and D-exo (Figures 2B). The size distribution of the Doxil-loaded exosomes (D-exo), measured by a nanoparticle tracking analyzer (Nanosight), was identical to those of non-manipulated exosomes (Figure 2C). The size of D-exo did not change significantly compared to the bare Doxil, indicating Doxil encapsulation and not simply superficial adhesion of Doxil to the exosomes. Flow cytometry was used to detect both the exosomal marker, CD81, and the fluorescence of doxorubicin, to verify that Doxil was successfully loaded into the exosomes (Figure 2D). Fluorescent microscopy confirmed that exosomes and Doxil were successfully fused, instead of aggregation (Figure S5). TUNEL staining confirmed the cytotoxic effects of D-exo. (Figure S6).

### Stability of D-exo in vitro

To assess the stability of D-exo over time, they were stored in phosphate buffered saline (PBS, 1x, pH 7.4) at 4°C for 7 days. To imitate the in vivo environment in which they will end up, they were placed in fresh serum at 37°C for 24 hours. NanoSight were employed to monitor the stability of D-exo in these two environments over time. The D-exo showed no obvious aggregation in PBS buffer or serum (Figures S7B-C), indicating superior stability. The drug-release behavior of D-exo was examined by monitoring doxorubicin released from the exosome at pH 7.4 (physiological environment) and 5.0 (late endosome) 13. D-exo released a small amount of doxorubicin at PH 7.4, which suggests that the drug was well protected by the exosomes in blood circulation. Conversely, a relatively rapid and massive release of doxorubicin was detected at PH 5.0, followed by a slow and continuous release thereafter (Figure S7D). Since the extracellular PH of tumor tissues is often acidic 14, these data suggest that drug was released promptly and substantially from D-exo after entering cancer tissues and late endosomes of cancer cells.

### Tumor uptake and inhibition

**Figure 3:**
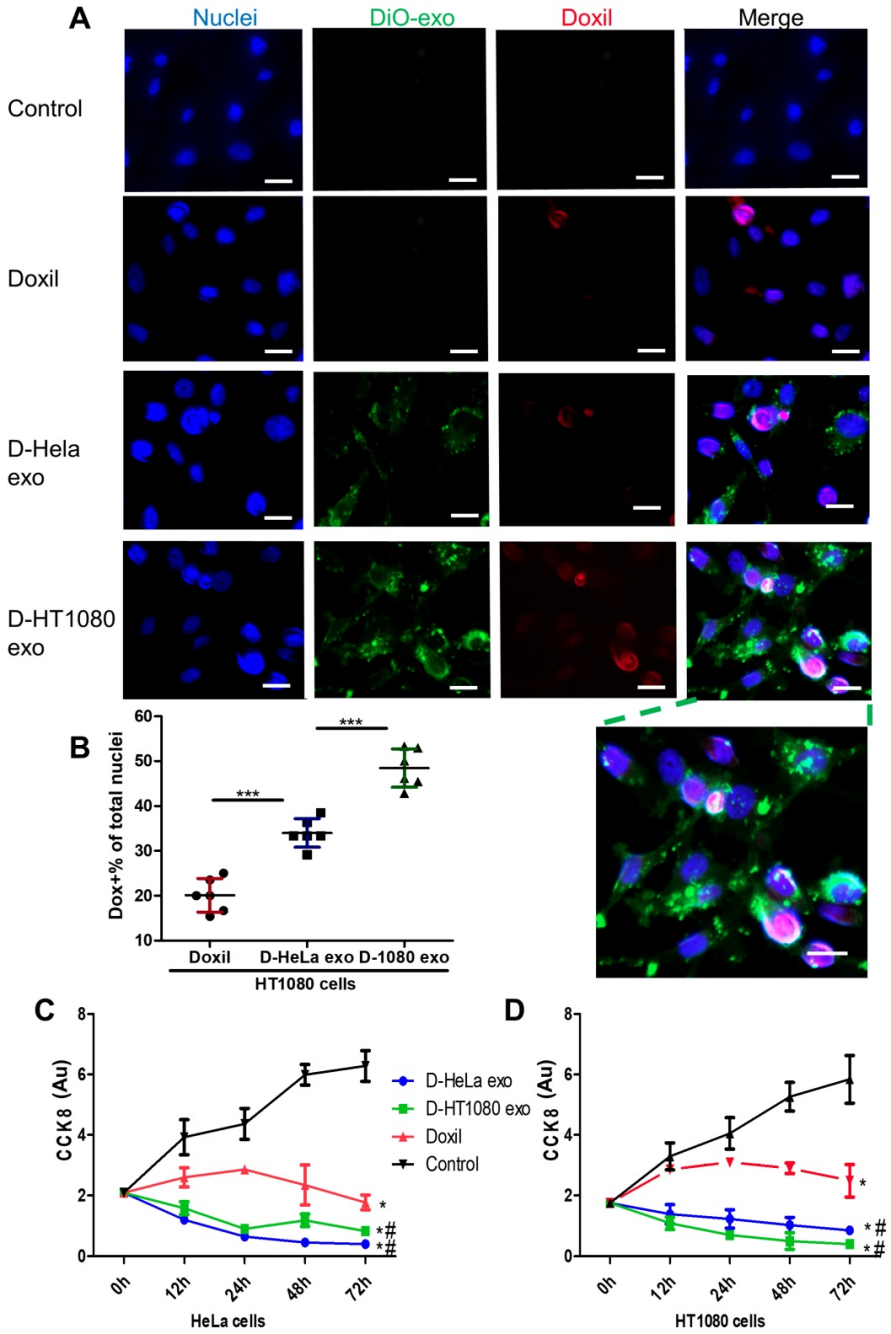
** Cancer cells uptake D-exo in vitro.** (A) Intracellular distribution of Doxil, D-HeLa exo, and D-HT1080 exo in HT1080 cells. Scale bar: 20µm. (B) Quantitation of the percentage of positive Doxorubicin in indicated treatment groups (n=6). One-way ANOVA with Bonferroni post hoc correction. ***, p<0.001. All values are mean ± S.D. (C-D) Cell viability of (C) HeLa cells or (D) HT1080 cells exposed to D-HeLa exo, D-HT1080 exo, Doxil, and PBS control (n=3). One-way ANOVA with Bonferroni post hoc correction. *, p<0.01 compared to control. #, p<0.05 compared to Doxil. All values are mean ± S.D.

DiO-labeled D-exo and free Doxil were incubated with HT1080 cells for 12 hours. As shown in Figure 3A, strong D-exo signals were dispersed within the cytoplasm, which suggests that D-exo were taken up by the cells. By 12 hours, doxorubicin had dissociated from the D-exo and distributed throughout the cytoplasm and nuclei of HT1080 cells. To estimate cellular uptake efficiency, D-exo labeled with DiO were incubated with HT1080 cells for 12 hours. The cellular uptake efficiency of Doxil from D-HT1080 exo, D-HeLa exo, and free Doxil were 48.46%, 34.00%, and 20.10%, respectively (Figure 3B). Although D-exo and Doxil are similar in size and morphology, cellular uptake of D-HeLa exo was nearly twice as high as that of Doxil, which indicates the preferential uptake of exosome-coated Doxil compared to bare Doxil. Moreover, HT1080 cells internalized D-HT1080 exo was twice as much as D-HeLa exo, indicating the cell-type tropism of cancer exosomes.

A Cell Counting Kit-8 (CCK-8) assay was used to assess the viability of HT1080 and HeLa cells in response to the growth-inhibition/cytotoxic effects of D-HeLa exo, D-HT1080 exo, or Doxil (with equivalent doxorubicin) and free exosomes. Exosomes did not induce any cytotoxic effects on cancer cells (Figure S8). As shown in Figure 3C, in accordance with the uptake efficiency results, D-HT1080 exo enhanced Doxil's suppressive effects on HT1080 cell viability. D-HT1080exo had the highest growth inhibition for HT1080 cells, while D-HeLa exo presented the lowest cell viability for HeLa cells. Collectively, these data suggested that cancer exosomes can shuttle anti-cancer drugs into the cancer cell types from which they were derived, thereby increasing the drug concentration in the cells to inhibit cells growth.

### Therapeutic effects of D-exo in vivo

**Figure 4:**
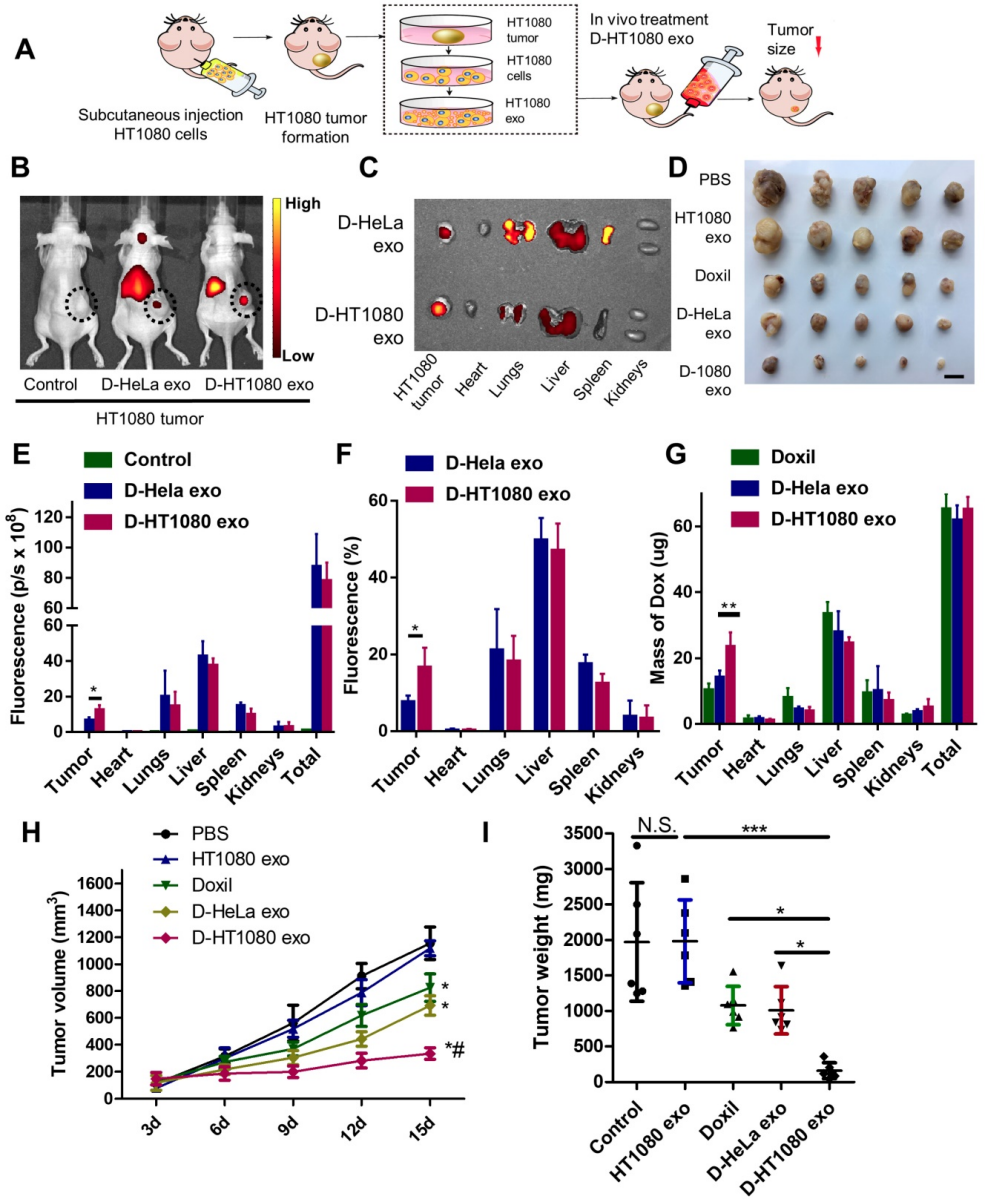
** Distribution and effects of D-exo in a mouse tumor model.** (A) Schematic showing the in vivo study design used to assess the effects of D-exo on nude mice bearing HT1080 tumors. (B) IVIS imaging of DiR-labeled D-exo in nude mice. (C) Representative ex vivo images of major organs and tumor tissues. (D) HT1080 tumor tissues obtained from indicated treatment groups. Scale bar: 1cm. (E) Quantitation of DiR-labeled D-exo in major organs and tumors (n=5). *, p<0.05. (F) Quantitation of the percentage of DiR-labeled D-exo in major organs and tumors (n=5). *, p<0.05. (G) Quantitation of Doxorubicin in major organs and tumor tissues (n=4). **, p<0.01. (H) Volume of HT1080 tumors in nude mice treated with PBS control, HT1080 exosomes, Doxil, D-HeLa exos, or D-HT1080 exos. Tumor volume was evaluated every 3 days for 15 consecutive days (n=6). *, p<0.01 compared to PBS control. #, p<0.05 compared to D-HeLa exo. (I) Mass of HT1080 tumors in nude mice with indicated treatment (n=6). *, p<0.05. N.S., no significance. (E-F) One-way ANOVA with Bonferroni post hoc correction. All values are mean ± S.D.

The cancer-targeting ability of D-exo was investigated using Nude mice bearing a subcutaneous HT1080 tumor as a model (Figure 4A). Firstly, we isolated HT1080 and HeLa cells from nude mice bearing HT1080 or HeLa tumors, then fabricated D-HT1080 exo and D-HeLa exo by collecting the respective exosomes released by each tumor and infusing them with Doxil. To gain insight into D-exo uptake sites in vivo, we intravenously injected D-HT1080 exo or D-HeLa exo, labeled with fluorescence probe DiR, into HT1080 tumor-bearing mice. We then used the IVIS imaging system to monitor the biodistribution of DiR-labeled D-exo. Only a slight fluorescence signal was detected at the tumor site in the mice treated with D-HeLa exo (Figure 4B), whereas D-HT1080exo yielded a significant fluorescence signal at the tumor site, which suggests that D-HT1080exo targeted the tumor more effectively than D-HeLa exo.

After in vivo imaging, the organs and tumor tissues of the mice were harvested for ex vivo imaging. Consistent with the in vivo imaging results, the fluorescent signal in tumors treated with Doxil-HT1080 exo was significantly higher than in those treated with D-HeLa exo (Figure 4C). The quantitative analysis revealed that the fluorescent intensity and the percentage of fluorescence in the D-HT1080 exo-treated tumor increased 1.9 fold and 2.2 fold, respectively, compared to the D-HeLa exo group (Figures 4E-F). These findings confirmed that D-HT1080 exo exhibited a tropism to accumulate at the HT1080 tumor site. In addition, the biodistribution of doxorubicin in the major organs and the tumor was examined (Figure 4G). Although Doxil and D-HT1080 exo were similar in size and morphology, they showed varied localization after injection. D-exo changed the biodistribution and enhanced the targeting efficacy of Doxil. D-HT1080 exo had the highest targeting efficacy. D-HT1080 exo increased the doxorubicin concentration 2.3-fold in the tumor compared to Doxil alone.

**Figure 5:**
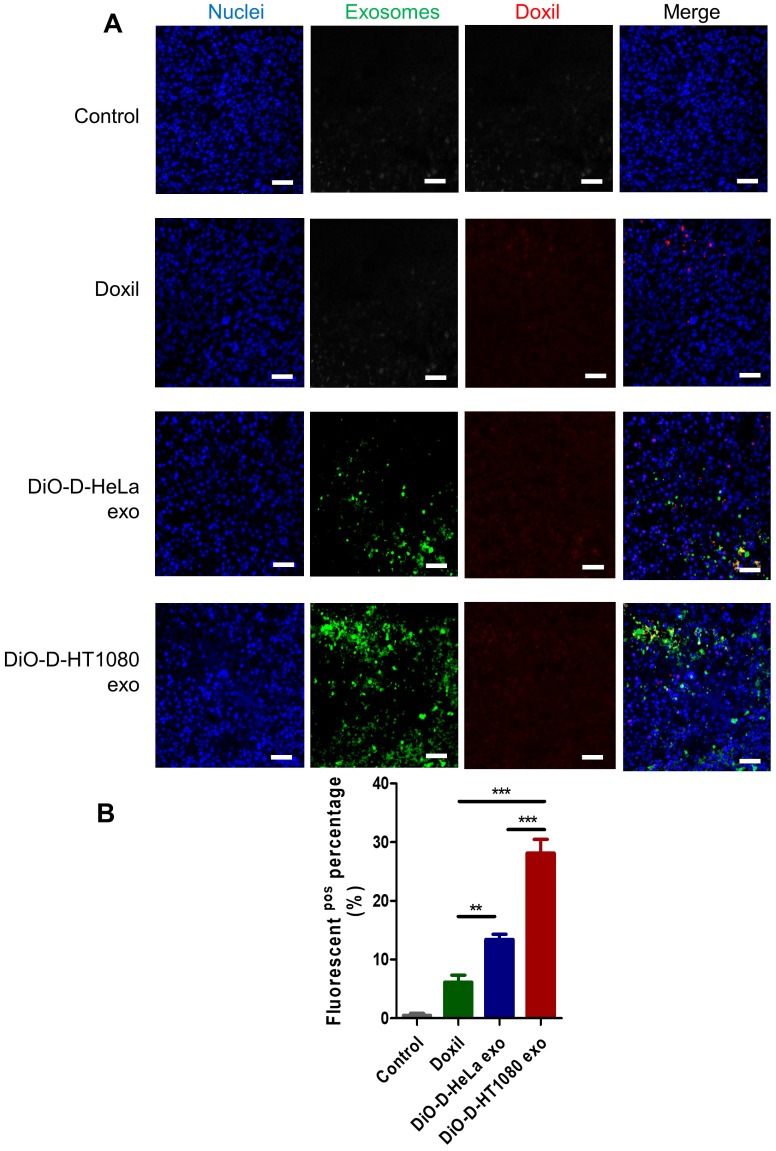
** Distribution of D-exo in tumor tissues.** (A) Representative micrographs showing accumulation of Doxil, DiO-labeled D-HeLa exo, and DiO-labeled HT1080 exo in tumor sections. Scale bar: 50µm. (B) Quantitation of Doxil, D-HeLa exo, and D-HT1080 exo in HT1080 tumor sections (n=3). **, p<0.01. One-way ANOVA with Bonferroni post hoc correction. All values are mean ± S.D.

To assess the distribution of D-exo and release of doxorubicin in tumor areas, we intravenously injected green (DiO) fluorescently labeled D-exo into nude mice and, 24 hours after injection, quantified their biodistribution and uptake in tumor tissues using fluorescent microscopy. HT1080 tumors absorbed much more D-HT1080 exo than D-HeLa exo or Doxil (Figures 5A-B). The fluorescent signals of D-exo (green) and doxorubicin (red) did not match completely because part of the doxorubicin was released from the D-exo 24 hours after administration. The data demonstrate that D-HT1080 exo effectively accumulated at the HT1080 tumor site and released doxorubicin.

To evaluate the capacity of D-exo to suppress tumors, mice with an HT1080 subcutaneous tumor were treated with repeated intravenous injections of D-HT1080 exo, D-HeLa exo, free Doxil, and free HT1080 exosomes in PBS (control group) every 3 days, for 15 days. In the treatment groups, every mouse received an equivalent dose of doxorubicin (5 mg doxorubicin/kg body weight) per injection. The tumor volumes of the control mice (free HT1080 exosomes and PBS) increased rapidly at an exponential rate. However, tumor growth was blunted with free Doxil and D-HeLa exo. Tumors treated with Doxil were less inhibited to grow than those treated with D-HeLa exo. By contrast, D-HeLa exo were more successful at suppressing tumor growth than Doxil. Moreover, while tumors treated with the control injections continued to grow, tumors that received D-HT1080 exo were reduced to nearly undetectable levels after 15 days of treatment (Figures 4H-I), Thus, the in vivo data indicates that HT1080 exosome injections result in enhanced doxorubicin accumulation at the cancer site compared with D-HeLa exo. D-HT1080 exo induced the most significant growth-suppression in HT1080 tumors, providing support for the above histopathological results, which indicate that D-HT1080 exo effectively accumulated at the HT1080 tumor site and release doxorubicin. Using Hematoxylin & Eosin (H&E) staining, no tumor formation was detected in any of the organs harvested from the mice that received HT1080 exosomes or D-HT1080 exo (Figure S9), confirming the viability of using HT1080 exosomes as a drug carrier in terms of safety.

Since D-HT1080 exo were manufactured by enveloping a Doxil sphere inside an HT1080 exosome membrane, we postulated that adhesion molecules on the exosome surface could play an important role in the homing phenomenon observed. Exosomes might express chemokine receptors that partner with chemokine ligands expressed in cancer cell membranes. Alternatively, both the exosomes and cancer cells could express the same receptors and bind to the same molecules in the local cancerous microenvironment (eg. extracellular matrix). We performed a proteome profiler array with 119 soluble receptors and related proteins on the cancer cells and exosomes. We identified 8 integrins among the top 40 most abundant soluble receptors, making integrins the most highly represented protein family in this analysis (Figure S10). These findings indicate a link between exosomal integrins and exosomal tumor tropism. Importantly, exosomal integrin expression does not necessarily reflect cellular integrin expression. This is due to the selective packaging of integrins in exosomes. Taken together, our data suggest that exosomal integrin expression patterns underlie the cancer exosome homing process.

## Discussion

Intercellular communication is essential for individual cell and multicellular organs to maintain homeostasis. Tumor cells continuously secrete membrane vesicles to the extracellular environment, among them, the tumor-derived exosomes contain specific repertoires of molecules, including DNAs, RNAs, lipids, and proteins, which are important for cancer cell communication with local cells and distant organs. Our findings suggest that exosomes derived from certain tumor cells can home to their parent tumor cell-type. This tropism is contrasted to the behavior of non-exosome-enveloped drugs and non-tumor-specific exosomes in vitro and in vivo in a mouse subcutaneous tumor model. Specifically, our tumor derived exosomes were engineered to carry Doxil, which is a sphere that encapsulates the cancer therapeutic, doxorubicin. In our study, tumor exosomes were able to effectively shuttle the chemotherapeutic to the tumor site, where it suppressed tumor growth.

Synthetic materials are difficult to recreate the biological functions of natural components which have evolved over time. Surface decoration with cancer cell membranes onto nanoparticles were frequently observed can evade the immune system to achieve highly specific targeting to tumors and efficient entry into cancer cells 15,16. Jingyi Zhu et al. created a magnetic iron oxide based nanoplatform that coated with cancer cell membranes, which achieves highly specific self-recognition to the source cell lines in vitro and excellent self-targeting homing ability to the homologous tumor in vivo even in the competition of another heterologous tumor 17. Ronnie H. Fang et al. coated cancer cell membrane onto polymeric nanoparticle cores made of poly (lactic-co-glycolic acid) (PLGA) polymer, the resulting cancer cell membrane-coated nanoparticle can be used to deliver tumor-associated antigens to antigen presenting cells or to homotypically target the source cancer cells 18. Wei Xie et al. loaded mesoporous silica nanoparticles with glucose oxidase and then encapsulated in cancer cell membranes, these synthetic complexes showed improved cancer targeting ability to ablate tumors and induce dendritic cell maturity to stimulate an antitumor immune response 19. Lang Rao et al. engineered a kind of cancer cell membrane-cloaked upconversion nanoprobes, which achieved highly specific in vivo tumor imaging 20. Hao Tian et al. fabricated cancer cell membrane camouflaged with hemoglobin and doxorubicin for homologous targeting and breaking hypoxia-induced chemoresistance 21.

Exosomes offer several advantages as carriers for cancer therapy 22-24. Exosomes originate in the endosomal compartment, the membranes of which contain both endosomal and plasma membrane proteins. Attempts have previously been made to develop exosome-based vesicles for the delivery of various therapeutic cargos, ranging from small-molecule chemotherapeutics and anti-inflammatory agents to miRNA and siRNA 25-27. Exosomes coating doxorubicin results in improving targeted tumor therapy and less drug accumulation in nontarget organs such as heart tissue and prevented off-target cardiotoxicity 28. Moreover, exosome encapsulation enhanced solubility for natural phytochemical compounds 29. Unlike liposomes and synthetic vesicles, exosomes express transmembrane and membrane-anchored proteins that may promote endocytosis, thus improving the delivery of their internal cargos 30. Moreover, exosomes contain integrin-associated transmembrane proteins that could activate the “don't eat me” signal that functions, in part, to protect them from phagocytosis 31, 32. Also, exosomes have excellent in vivo stability and less cytotoxicity than synthetic drug carriers 33. In this study, we use Doxil instead of non-encapsulated doxorubicin because of Doxil's similarities in size and morphology to exosomes. The congruence between the two make Doxil an excellent control for the exosomes used in this study. Moreover, our data demonstrate that when Doxil is loaded into exosomes derived from their parent tumor, they target and bind said tumor to a greater extent than Doxil encapsulated in other cancer cell lines.

Though the mechanism of the homologous exosome targeting to cancer cells remains unclear, it has been demonstrated that tumor cells readily agglomerate to solid tumors contributing to the surface proteins with homophilic adhesion domains such as integrin, focal adhesion proteins, and RHO family proteins 34, 35. Current cancer targeting is achieved largely by receptor-ligand interactions on the specific surface antigens on cancer cells 36, 37, or by coating nanoparticles in cancer cell membrane to take advantage of the inherent cell-to-cell adhesion property of cancer cells 17-21. Recently, nanoparticles were cloaking with red blood cell membranes or cancer cell membranes for long blood-circulation time and were capable of evading the immune system by the mechanisms of immune tolerance, immunosuppression, and immunosenescence 38. The mechanism by which exosomes are absorbed by the receiving cancer cells remains unclear. Isabella et al. showed that cancer exosomes fuse with parental cells through lipid mixing. The lipid composition of tumor cell membranes is a key factor in determining exosome to tumor cell fusion 10. Tyson et al. showed that besides lipid composition, the unique protein composition of exosomes also facilitates the internalization of exosomes in cancer cells 39. Ayuko et al. reported that unique exosomal integrins interact with cell-associated extracellular matrix (ECM), mediating exosomal uptake in the cell of specific target organs. For example, exosomes expressing integrin α6β4 and integrin α6β1 bind to lung-resident fibroblasts and epithelial cells in laminin-rich lung microenvironments, driving lung tropism in these exosomes 40. Bong Hwan et al. revealed that exosomes play a critical role in the adhesion assembly of cancer cells, which promotes cell motility. A primary driver of said motility is fibronectin, which is an exosome cargo that uses integrin binding to sort into the exosomes 41. Our proteomic results demonstrate that integrin might play a role in the adherence of exosomes to the recipient cancer cells. The cancer cells we screened share a number of integrins with their secreted exosome. Those exosomes adhere to specific ECM molecules (such as fibronectin, laminin, or collagen fibers) that facilitate their homing to the parent tumor tissues.

Contrary to our report, Tyson et al. reported a rapid clearance and minimal accumulation of intravenously-injected exosomes and liposomes in their tumor models. They also reported a similar accumulation of exosomes in their parent cell lines and in cells of various other origins 39. A possible explanation for the observed differences between our results and those of Tyson et al. is the exosome source. We transplanted the cancer cell lines in this study into nude mice to induce mouse tumors. Then, we harvested tumor cells from the mice models and isolated exosomes from these harvested cells. It is possible that exosomes derived from different cancer cell lines have different homing capabilities. In addition, the dosage and injection frequencies might affect the biodistribution of exosomes in vivo 42. Tyson et al. injected their exosomes once at a dosage of 60 ug. We injected about 10^10^-10^11^ particles every 3 days for a total of 5 injection. Thus, our dose was about 5 times higher than that of Tyson's group.

One of the side effects of Doxil is cardiac toxicity, which puts a limit on the maximum amount of Doxil that can be administered in cancer patients. The drug concentrations in the heart of mice treated with D-exo were much less than in those treated with Doxil. In other words, D-exo reduce the nonspecific distribution of exosomes in the heart, extending the maximum amount of Doxil available to cancer patients and reducing cardiotoxicity.

Using natural cell membrane to coat nanoparticles provides a promising method to improve nanoparticle biocompatibility. Most of these studies used cancer cell membrane to cloak polymeric nanoparticles loaded with small molecule drug, siRNA, or photosensitizer, the cancer membrane-coated nanoparticles have proven effective for immune escape and homotypic targeting attributed to the presence of specific surface antigens on cell membranes 43. Red blood cell membrane, white cell membrane, and platelet membrane enveloping nanoparticles have also been demonstrated to improve the nanoparticle functionality by mimicking the properties of source cells 44-46. More recently, studies showed that in comparison to cell membranes, cell-derived exosomes could be used as better membrane materials to fabricate biomimetic nanoparticles. Membrane protein can protect exosomes from clearance by the mononuclear phagocyte system and increase the stability in circulation. Homotypic adhesion molecules on exosome membrane also endow exosomes with strong preferential binding to source cells. Therefore, exosome membrane-coated nanoparticles could offer significant advantages in terms of prolonged blood circulation time, optimal biocompatibility, and enhanced targeting effect 47. The novelty of this study are as follows: Firstly, tumor-associated antigens such as monophosphoryl lipid A(MPLA) 18, 5- hydroxytryptamine (5-HT) 48 were used in the previous studies to promote homing to tumor and permits sensitive discrimination between cancerous and noncancerous cells. Secondly, they aimed to compare the therapeutic index of exosome-coated nanoparticles to cancer membrane-coated nanoparticles or lipid membrane-coated nanoparticles, so the controls they used were cancer membranes or lipid membranes. Thirdly, the antitumor payload they used to load into exosomes were doxorubicin, siRNA, apoptosis-inducing proteins et.al. Doxil is the first time been used to load into exosomes.

## Conclusions

In conclusion, our findings suggest that cancer-derived exosomes have the ability to home to their parent tumors and that drug-loaded cancer exosomes can be used for targeted cancer therapies. Despite these promising results, using cancer-derived exosomes in cancer treatment should be done with caution due to their described metastatic role in tumor progression. Future studies will focus on identifying exosomal integrins, other proteins, or lipids that play key roles in the exosome homing mechanism. Another focus will be the identification of the types of cells (fibroblasts, endothelial cells, perivascular cells, or inflammatory cells) that receive the exosomes in the tumor tissues. Our findings indicate that exosomes derived from cancer patients might be a good weapon in the fight against cancer.

## Methods

### Cell culture, exosome isolation, and labeling

The HeLa and HT1080 cell lines were purchased from Sigma-Aldrich (ECACC, 93021013 and 85111505). Cells were cultured until 80% confluency and washed 3 times with DMEM. Then, the medium was switched to DMEM. They were then conditioned for 48 hours, after which exosomes were isolated as previously described11,12. In brief, exosomes were isolated by ultrafiltration. Conditioned medium was filtered through 0.22 µm Steriflip filters to remove cellular debris and large vesicles. The filtrate was then added to Amicon Ultra-15 100 kDa filters (Millipore, SCGP00525) to centrifuge at 5,000 × g for 5 min. The flow-through was discarded and the concentrated exosomes were collected and washed with PBS three times before being stored at -80°C.

Exosome labeling was performed using 10 uM DiI or DiO (Thermo Fisher, V22889), incubated for 20 minutes at 4°C. Then, Exosome Spin Columns (Thermo Fisher, 4484449) were used to remove the unincorporated dye.

### Cancer exosome homing to the parent cell line in vitro

HT1080 or HeLa cells were seeded at a density of 2 × 10^4^ cells/mL in 4-well culture slides. After culturing for 24 hours, the culture medium was replaced with DMEM, containing 7 × 10^8^ DiI or DiO-labelled HT1080 exosomes or HeLa exosomes. Cells were cultured for an additional 12 hours, washed three times with PBS, fixed with 4% paraformaldehyde, and stained with DAPI (Life Technologies, R37606). Images were taken with a fluorescent microscope (Olympus, Olympus IX81).

HeLa cells and HT1080 cells were seeded at a density of 2 × 10^4^ cells/mL in 96-well culture plates. After culturing for 24 hours, the culture medium was replaced with DMEM, containing 1.75 × 10^8^ DiI-labelled HeLa exosomes or HT1080 exosomes, for 12 hours. A Fluoroskan Ascent™ FL Microplate Fluorometer (Thermo Fisher, 374 Fluoroskan Ascent™ FL) was used for fluorometric measurements.

### Cancer exosome homing to the parent cell line in vivo

100 µl of DMEM, containing 2 × 10^6^ HT1080 cells, were mixed with 100 µl of PBS and subcutaneously injected into the flanks of immunocompromised Nude mice (Charles River Labs, Wilmington, MA). After two weeks, the tumors had grown to about 200 mm3. At that point the mice were divided randomly into three groups: (1) Intravenously injected with 3 × 10^11^ DiR-labeled HT1080 exosomes in 100 µl of PBS. (2) Intravenously injected with 3 × 10^11^ DiR-labeled HeLa exosomes in 100 µl of PBS. (3) 100 µl PBS. After 24 hours, the mice were euthanized and the tumors and major organs were harvested. An IVIS Spectrum imaging system (Caliper Lifesciences) was used to detect the fluorescence in each organ.

### Fabrication and characterization of D-exo

To detect the highest yield of Doxil-loaded Exosomes, we loaded Doxil into exosomes at different concentrations (10^10^,10^11^,10^12^,10^13^). We then incubated the reaction mixture at different temperatures (4°C and 25°C) for 30 min. The highest yield was attained at a concentration of 10^11^ exosomes, reacted at 4°C. Then, Doxil-loaded exosomes were fabricated by extruding through membranes with pore sizes of 200 nm using an extruder (Avanti Polar Lipids, 610000). The excess Doxil was removed by filtering through an Amicon Ultra-15 100 kDa filter. The amount of Doxorubicin loaded into exosomes was measured from a calibration curve obtained through colorimetric measurements, using a microplate reader (Tecan Sunrise, F039300) at an absorbance wavelength of 485 nm.

Nanoparticle tracking analysis (Malvern, NanoSight NS300) was used to measure the concentration and size distribution of exosomes and D-exos. Each sample was imaged five times for 60 seconds and analyzed. Successful exosome coating was confirmed with confocal laser scanning microscope (Carl Zeiss, Zeiss LSM 710) and transmission electron microscopy (JEOL, JEM-2000FX electron microscope). Flow cytometry was used to characterize the exosomal marker, CD81, and the fluorescence of Doxorubicin, to verify that Doxil was successfully loaded into the exosomes.

Drug release was performed under pH 7.4 (PBS) and PH 5.0 (acetate buffer). In short, 1 mL of D-exo solution was added into an Amicon Ultra-15 3K filter (Millipore, UFC900324). The filter was placed into a collection tube with 5 mL of PBS or acetate buffer. The release of doxorubicin was performed at 37°C for 48 hours. At selected time intervals, the collection tube was replaced with a new one. The doxorubicin concentration was detected through colorimetric analysis, using a microplate reader (Tecan Sunrise, F039300), at a detection wavelength of 485 nm. The concentration was calculated based on standard curves of doxorubicin solution at different PH values.

To measure the storage stability of D-exos, they were incubated in glass vials at 4°C in PBS. To measure their application stability, they were incubated in glass vials at 37°C in serum. At selected time intervals, the particle concentration and size distribution of the two samples were evaluated using the NanoSight.

### Tumor cells uptake D-exo in vitro

HT1080 cells were seeded at a density of 2 × 10^4^ cells/mL in 4-well culture slides. DiO-labeled D-HT1080 exo, DiO-labeled D-HeLa exo, Doxil, and PBS were co-cultured with the HT1080 cells for 12 hours respectively. The slides were washed three times before staining with DAPI. Images were taken on an Olympus IX81 fluorescent microscope and analyzed with NIH Image J software.

### Tumor cell growth-inhibition assays in vitro

The effect of exosomes or D-exo on cancer cells was evaluated by a Cell Counting Kit-8 (CCK-8, Sigma-Aldrich, 96992). First, 2 × 10^4^ cells/mL HeLa or HT1080 cells were seeded into 96-well plates and grown in DMEM medium containing 10% fetal bovine serum for 24 hours. Then, the culture medium was replaced with fresh medium containing HeLa exosomes, HT1080 exosomes, D-HeLa exos, D-HT1080 exos, Doxil, or PBS, and cultured for 12h, 24h, 48h, 72h respectively. D-HeLa exos, D-HT1080 exos, and Doxil each carried the same concentration of Doxorubicin (5µg/mL). Then CCK-8 solution was added and cell viability was measured using a microplate reader (Tecan Sunrise, F039300) at 450 nm.

### D-exo biodistribution and targeting for cancer in vivo

100 µl DMEM, containing 2 ×10^6^ HT1080 cells or HeLa cells, were injected subcutaneously into the flanks of 4-week-old Nude mice. After two weeks, the tumors had grown to about 200 mm^3^. HT1080 or HeLa tumor tissues were harvested from the inner side of the tumors, cut into small fragments, and enzymatically digested with collagenase. The digested mixture was diluted with DMEM, containing 10% FBS, then filtered through a 40-µm sterile cell strainer. The filtered cells were collected and expanded for future exosome isolations. D-HT1080 exos and D-HeLa exos used for in vivo treatments were generated from these exosomes.

HT1080 tumor studies were organized as described below. The HT1080 tumor-bearing mice were divided randomly into four groups: (1) Intravenously injected with DiR-labeled D-HT1080 exos in 100 µl of PBS. (2) Intravenously injected with DiR-labeled D-HeLa exos in 100 µl of PBS. (3) Intravenously injected with Doxil in 100 µl of PBS. (4) Intravenously injected with 100 µl of PBS. Each treatment group used the equivalent dose of doxorubicin (5 mg of doxorubicin per kg of body weight). Treatments were performed every 3 days for 15 days. In vivo imaging was recorded using an IVIS Spectrum imaging system. Subsequently, the mice were euthanized and the tumors and major organs were harvested for ex vivo imaging.

To investigate the biodistribution of doxorubicin released by D-exo, the harvested tumors and major organs were stored in liquid nitrogen and ground in a mortar. The powder was then dissolved in 1 mL of borate buffer solution and lysed using ultrasonication. After 30 min, 1 mL of chloroform was added to the solution, which was then agitated for 30 min. Finally, the solution was allowed to remain stationary and separate out. The lower, denser layer was collected and tested using a colorimeter. According to the absorption standard curve of doxorubicin, the absorbance was measured at 485 nm to determine the doxorubicin content.

At the end of the treatment, the mice were euthanized, and the tumors were harvested. Mouse weights, tumor mass, and tumor volumes were measured. The tumor volume was measured with a caliper and calculated as length × width^2^/2.

### Histology

To observe the biodistribution of D-exo and doxorubicin in tumor tissues, DiO-labeled D-exo solution (5 mg/mL; 200 μL per mouse) was injected intravenously into mice with cancer. There were four groups: (1) Dio-labeled D-HT1080 exo, (2) Dio-labeled D-HeLa exo, (3) Doxil, and (4) PBS control group. After 24 hours, tumor tissues were harvested and embedded in optimal cutting temperature (OCT) compound, snap-frozen in liquid nitrogen, and then cryosectioned at a thickness of 10 µm. Tumor sections were then fixed in 4% paraformaldehyde solution, treated with DAPI, and preserved in Prolong Gold Mountant. The images were taken with an Olympus IX81 fluorescent microscope.

For the histopathological evaluation of major organs, they were harvested and embedded in OCT, snap-frozen in liquid nitrogen, and then cryosectioned at a thickness of 10 µm. Hematoxylin and Eosin staining (H&E) was performed to further confirm the absence of tumors in all harvested organs.

### Exosome proteome array analysis

The proteins of cancer cells and exosomes were extracted with RIPA lysis buffer (Sigma, R0278). Total protein was measured with a BCA Protein Assay Kit (Life Technologies, 23225). Protein arrays were detected using a receptor protein array (R&D systems, ARY012) according to the manufacturer's instructions.

### Statistics

GraphPad Prism (GraphPad Software, La Jolla, CA) was used for statistical analysis. Results were presented as mean ± S.D. All comparisons between two groups were performed with two-tailed unpaired Student's t-test. One-way ANOVA analysis, with post hoc Bonferroni correction, was used to compare means among more than two groups. Differences were considered statistically significant when p < 0.05.

## Supplementary Material

Supplementary figures.

## Abbreviations

D-exo: Doxil-loaded exosomes
D-HeLa exo: Doxil-loaded Hela exosomes
D-HT1080 exo: Doxil-loaded T1080 exosomes
ECM: extracellular matrix
TEM: Transmission electron microscopy
