# Supplementary material for: Tumor cell-derived exosomes home to their cells of origin and can be used as Trojan horses to deliver cancer drugs
Source: Theranostics. 2020 Feb 10;10(8):3474–87. doi: 10.7150/thno.39434 (PMC7069079; doi:10.7150/thno.39434)
Supplement: Supplementary file 1 — Supplementary figures. [file thnov10p3474s1.pdf]

Supplemental data

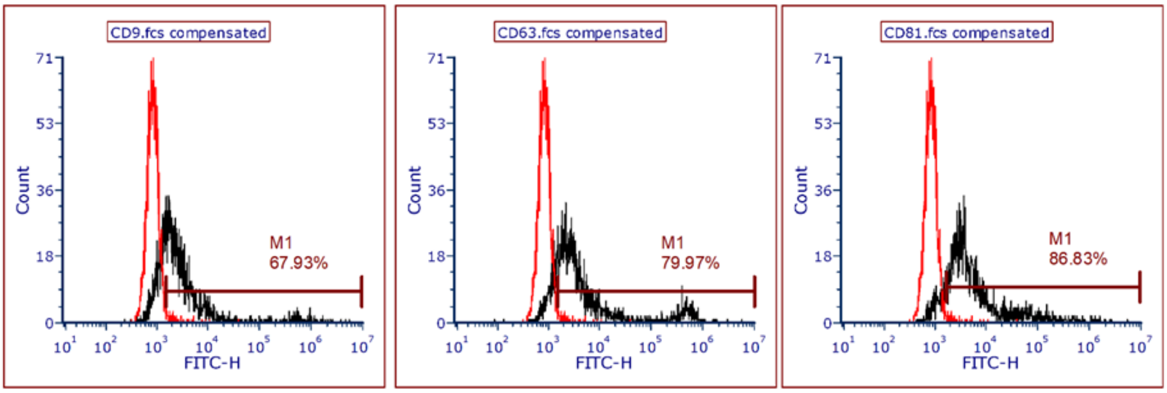

Fig. S1| Flow cytometry showing exosomal markers, CD81, CD63, and CD9 were positively expressed on exosomes that we used.

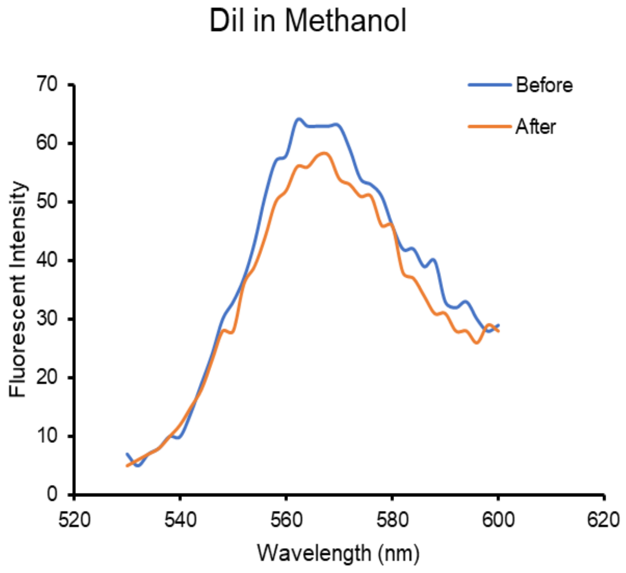

Fig. S2| Fluorescent intensity of solution (diluted in methanol) before and after spin columns. 10 µL DiI diluted to 1 mL methanol. The labeling efficiency is higher than 90%.

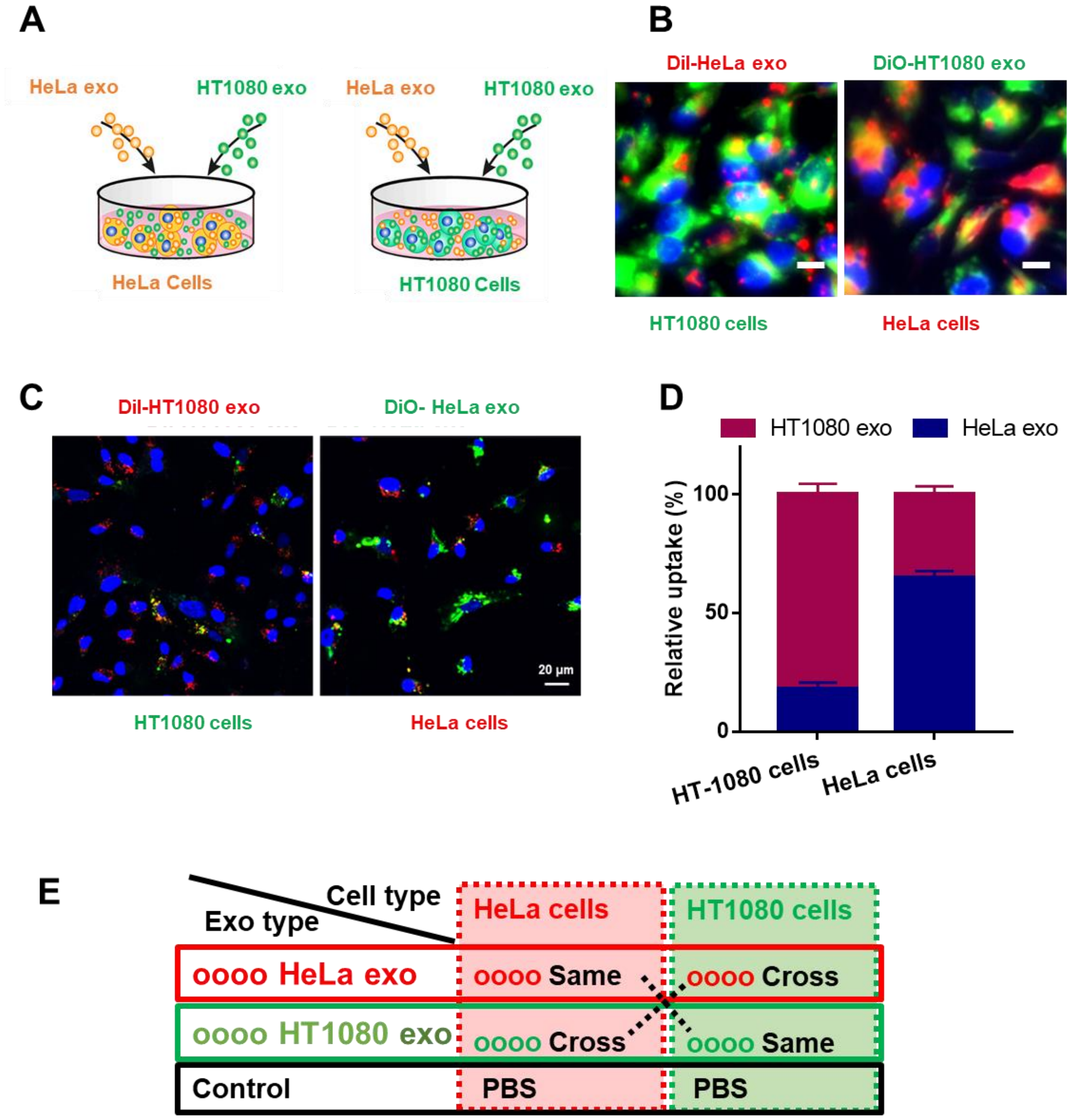

Fig. S3| Cancer cell-derived exosomes preferentially localize to their parent cells in vitro. (A) Schematic showing the *in vitro* study design to evaluate the capacity of cancer exosomes to target their parent cells. (B) Representative micrographs showing uptake of DiI-labeled HeLa exosomes (red) and Dio-labeled HT1080 exosomes (green) by HeLa cells or HT1080 cells. Scale bar: 5μm. (C) Representative micrographs showing uptake of DiO-labeled HeLa exosomes (green) and DiI-labeled HT1080 exosomes (red) by HeLa cells or HT1080 cells. Scale bar: 20μm. (D) Quantitation of exosomal uptake in HeLa cells and HT1080 cells (n=3). (E) Schematic showing the in vitro study design to assess the homing ability of cancer exosomes by Microplate Fluorometer.

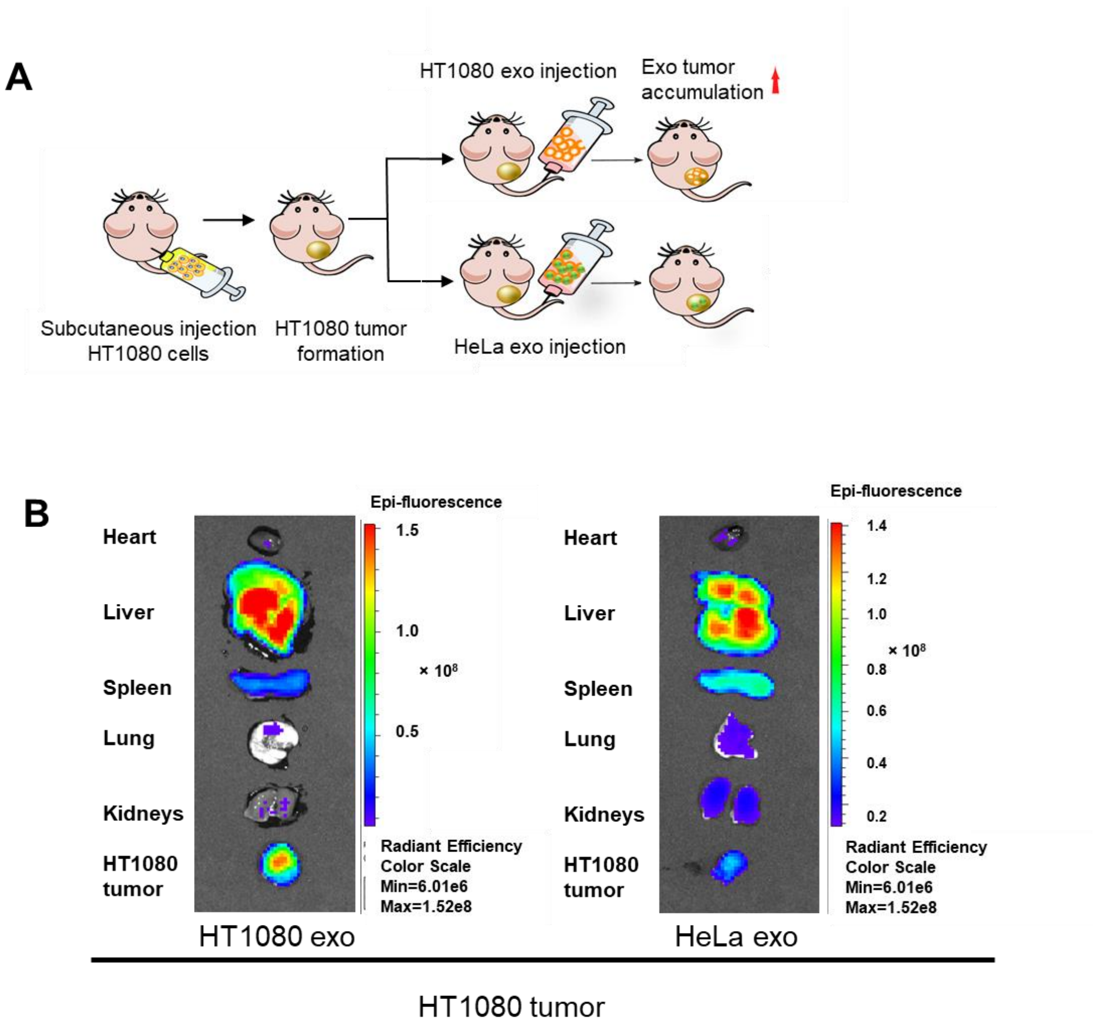

Fig. S4| Cancer cell-derived exosomes preferentially localize to their parent cells in vivo. (A) Schematic showing the in vivo study design to assess the homing ability of cancer exosomes in a nude mouse subcutaneously bearing HT1080 tumors. (B) Representative ex vivo fluorescent imaging of major organs and tumor tissues 12 hours after exosomes injection.

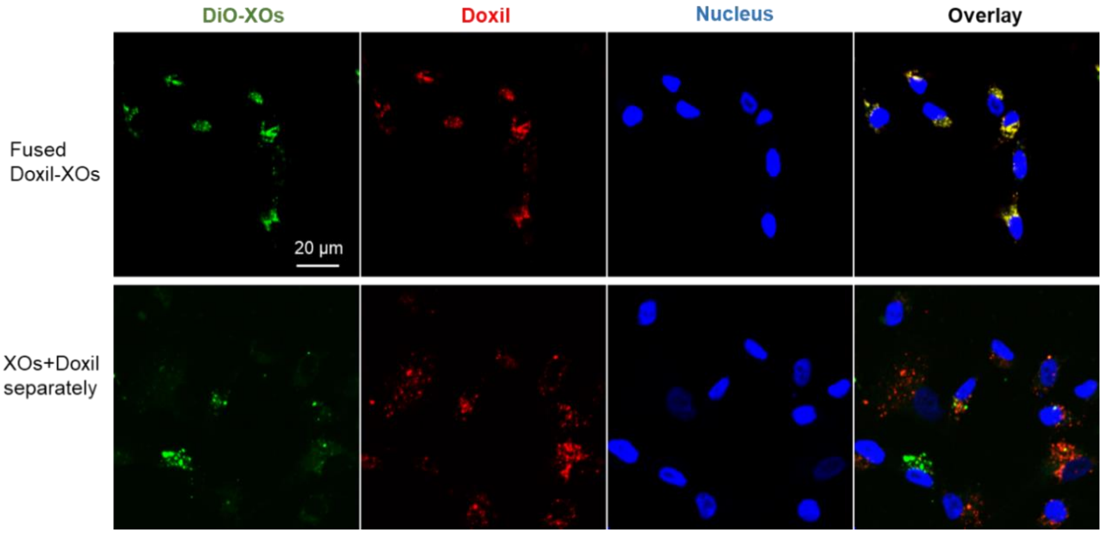

Fig. S5| Representative fluorescent images showing DiO-labeled fused Doxil-exosomes (D-exo) and separated exosomes+Doxil. We used DiO to label exosomes (green) and prepared fused doxil-XOs. Fused doxil-exosomes will be yellow after uptake, while signals of exosomes and doxil were separate when added separately.

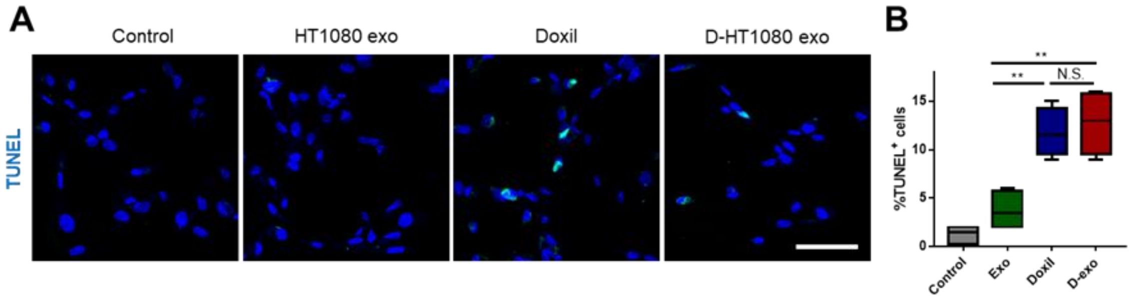

Fig. S6| Cytotoxic effects of D-exo. (A) Apoptotic HT1080 cells in response to PBS, HT 1080 exo, Doxil or D-HT1080 exo treatment. Scale bar: 100 µm. (B) Quantitation of apoptotic cells (n = 6).

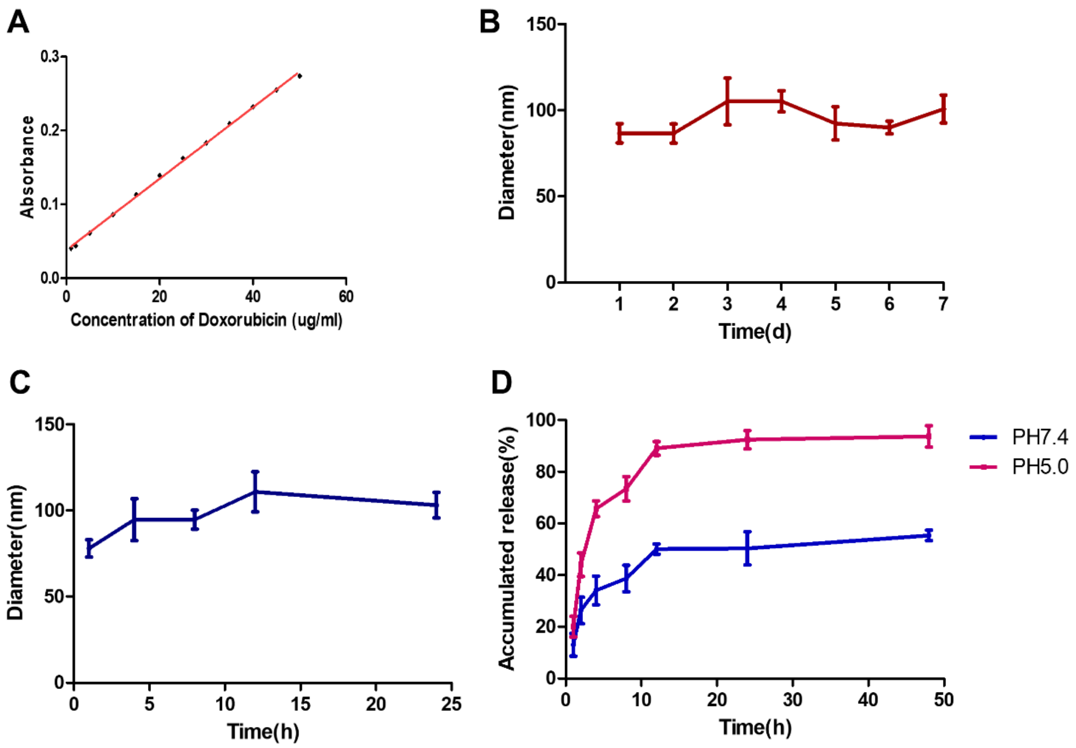

Fig. S7| Characterization of D-exo. (A) Standard curve of Doxorubicin concentration. (B) Change in size of D-exo stored at 4°C in PBS for 7 days (n=5). (C) Change in size of D-exo stored at 37°C in serum for 24 hours (n=5). (D) Release profiles of Doxorubicin from D-exo in PBS (PH=7.4) and acetate buffer (PH=5.0) (n=3). All values are mean ± S.D.

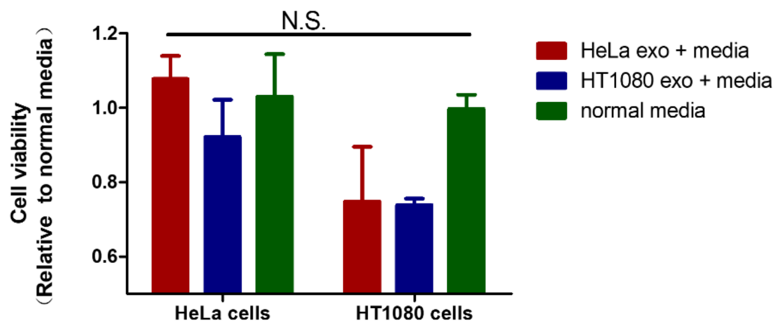

Fig. S8| Effect of cancer exosomes on cancer cell viability. (A) Cell viability of HeLa and HT1080 cells treated with HeLa exosomes or HT1080 exosomes (n=6). Two-tailed t-test. N.S., no significance. All values are mean ± S.D.

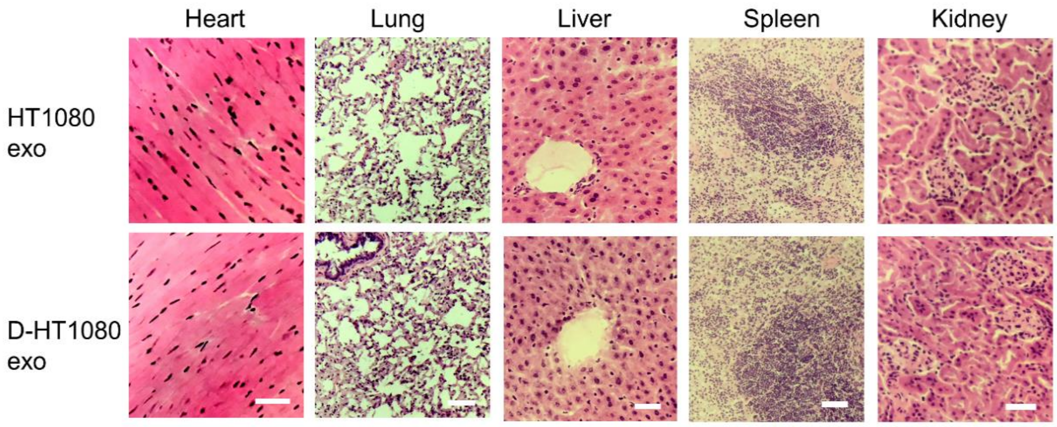

Fig. S9| Histological analysis of major organs. (A) Histological sections of major organs stained with hematoxylin and eosin. No tumor formation was detected in any harvested organ. Scale bar: Heart=30um, Lung=50um, Liver=30um, Spleen=50um, and Kidney=30um.

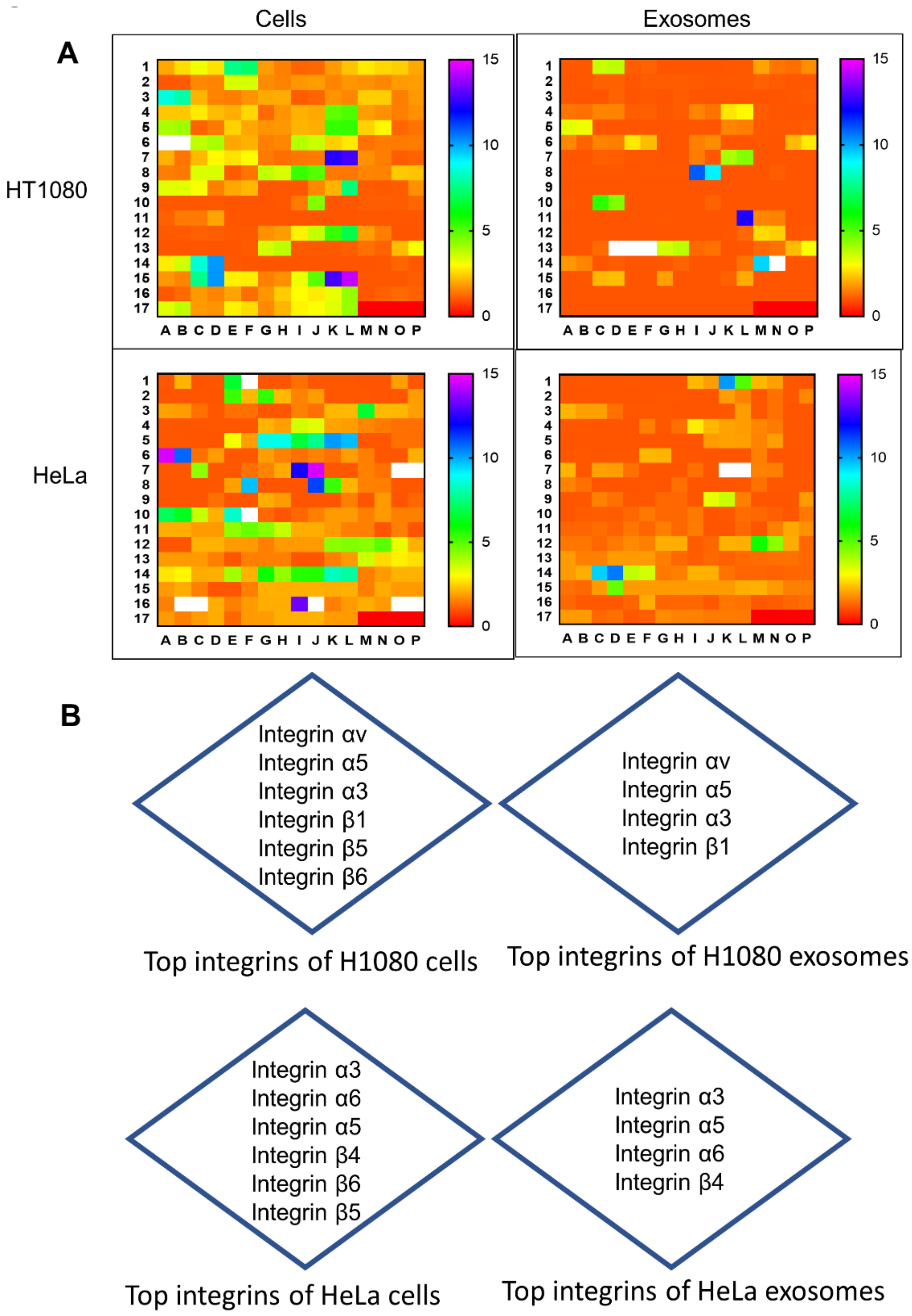

Fig. S10| Heat map of the receptor proteome profiler array identifying integrins that might contribute to the tumor exosome homing mechanism. (A) Heat map showing fold changes between tumor cells and exosomes. (B) Top integrins in HT1080 or HeLa cells and exosomes (n=4).
